# Gardening in the zone of death: an experimental assessment of the absolute elevation limit of vascular plants

**DOI:** 10.1038/srep24440

**Published:** 2016-04-13

**Authors:** Miroslav Dvorský, Zuzana Chlumská, Jan Altman, Kateřina Čapková, Klára Řeháková, Martin Macek, Martin Kopecký, Pierre Liancourt, Jiří Doležal

**Affiliations:** 1 Institute of Botany, The Czech Academy of Sciences, Třeboň, Czech Republic; 2 Department of Botany, University of South Bohemia, České Budějovice, Czech Republic; 3 Department of Ecosystem Biology, University of South Bohemia, České Budějovice, Czech Republic; 4 Institute of Botany, The Czech Academy of Sciences, Průhonice, Czech Republic; 5 Department of Botany, Faculty of Science, Charles University, Benátská 2, Praha, CZ-128 01, Czech Republic; 6 Department of Vegetation Ecology, Institute of Botany, The Czech Academy of Sciences, Brno, Czech Republic; 7 Department of Forest Ecology, Faculty of Forestry and Wood Sciences, Czech University of Life Sciences Prague, Kamýcká 129, CZ-165 21, Praha 6-Suchdol, Czech Republic

## Abstract

Vascular plants in the western Tibetan Plateau reach 6000 m-the highest elevation on Earth. Due to the significant warming of the region, plant ranges are expected to shift upwards. However, factors governing maximum elevational limits of plant are unclear. To experimentally assess these factors, we transplanted 12 species from 5750 m to 5900 m (upper edge of vegetation) and 6100 m (beyond range) and monitored their survival for six years. In the first three years (2009–2012), there were plants surviving beyond the regional upper limit of vegetation. This supports the hypothesis of dispersal and/or recruitment limitation. Substantial warming, recorded *in-situ* during this period, very likely facilitated the survival. The survival was ecologically a non-random process, species better adapted to repeated soil freezing and thawing survived significantly better. No species have survived at 6100 m since 2013, probably due to the extreme snowfall in 2013. In conclusion, apart from the minimum heat requirements, our results show that episodic climatic events are decisive determinants of upper elevational limits of vascular plants.

Distribution of many species in alpine regions has been documented to be shifting as a result of the ongoing climate change^1^,^2^,^3^,^4^,^5^. Alterations of warming and precipitation regimes affect the performance of species particularly at their range margins. Alpine plants are particularly sensitive to climate change so high mountains provide suitable natural laboratories for tracing climate-induced biological responses^6^. In alpine regions, climate change may ameliorate growing conditions for plants and, thus, may open unpopulated habitats. The pace of colonisation will depend on the dispersal capacities of the upper populations of species. When climate change leads to the deterioration of conditions, alpine perennial plants may still persist as they are capable of surviving in apparently suboptimal conditions for considerable periods of time, even a few decades^7^. The net effect of climate change is, however, more complex and likely combines an amelioration of some aspects of the environmental conditions and the deterioration of others. For example, a period of warmer summers may support generative reproduction and result in establishment of populations at higher elevations, while longer and more snowy winters may eliminate them, especially at their uppermost ecophysiological limits^8^.

The highest elevation limit of of continuous, although sparsely scattered, vegetation in the world has been reported from the westernmost part of the Tibetan Plateau, and it is located at ca. 6000 m^8^,^9^. This part of the Transhimalaya, i.e. the arid land north of the main Himalaya Range, provides the most suitable conditions for plant occurrence at extreme altitudes^10^. The factors facilitating its habitability include (1) favourable geomorphology (gentle slopes of the plateau preventing serious erosion and substrate instability), (2) arid climate (<100 mm yr^−1^; preventing extensive glaciation; the snow line located hundreds of meters above the vegetation limit; limited frost-heave and solifluction in dry substrates), (3) suitable geology such as metamorphic bedrock producing relatively fine substrates at a limited occurrence of coarse screes, and (4) location within a subtropical belt (high solar input). All of these conditions predominate over the whole south and west Tibetan Plateau (which stretches to East Ladakh), where even higher limits of continuous vegetation can be expected. To the best of our knowledge, such higher limits have not been reported so far. The highest record was reported from the south of the Tibetan Plateau, from the region north of Mt. Everest^11^ which already lies in the rain shadow of the main Himalaya Range. This record of *Saussurea gnaphalodes* at 6400 m originates from the 1930s and has never been confirmed since; apart from the doubts about the correctness of the elevation measurements and the absence of microclimatological characterisation of the site, records like this tell little about the elevation limits of continuous vegetation on Earth.

Climate change in Ladakh, as well as the rest of the western Himalaya and the adjacent part of the Tibetan Plateau, includes undoubted rapid warming^12^,^13^,^14^. The rate of warming may be different at higher elevations^15^, e.g. in Tibetan Plateau it does not continue to rise above 4800 m and remains at a high level with a slight decline at the highest elevations^16^. Warming leads to an extension of the growing season due to earlier snowmelt and later snowcover, and helps to expose colonizable substrates, e.g. after glacier retreat^17^. These phenomena enable vegetation to expand beyond its range. Precipitation change in the region exhibits a more local pattern and a study explicitly concerning our study region is not known to us. In the surrounding area, where studies have been made, there is mainly an indication of increased precipitation^13^,^14^,^18^,^19^,^20^,^21^,^22^, although reports of a decrease have also been made^23^. The increasing trend of summer and winter precipitation over the Himalaya is associated with an increasing trend in precipitation extremes connected to the changing pattern of the summer monsoons^20^,^24^,^25^,^26^,^27^, and an enhancement in the strength and frequency of winter westerly disturbances^18^,^28^,^29^,^30^,^31^ (extratropical storms driven by the Westerlies, that bring sudden winter rain and snow to the northwestern parts of the Indian subcontinent), respectively. Indeed, over the last decade, Ladakh experienced unusual summer storms, causing massive flash floods^32^. In addition, years 2013 and 2015 have had the most extreme snowfall in the last 50 years.

In the region of study, the subnival zone of Eastern Ladakh, snowfall occurs all year round. An increase in precipitation may cause thicker snow cover which potentially leads to a delayed disappearance of snow, shortening of the vegetation season, enlargement of the permanent snowfields and the eventual concealment of habitable places. Wetter substrates are also more susceptible to frost heave, a serious factor complicating seedling establishment and even the persistence of mature plants^6^ adapted to the arid substrates of cold, high-altitude deserts. Thus, the factors associated with higher water input into the ecosystem can make the survival at the extreme elevations harder^33^ and, in the long term, may contribute to the shrinkage of the subnival zone.

As the effect of increased precipitation on the direction of the vegetation shift may be opposite to the effect of warming, the principal questions are whether the current upper species ranges are (1) in an equilibrium state with the current climate, (2) lagging behind the climate amelioration (warming) due to dispersal limitations, i.e. there are uninhabited habitats available beyond the current range, or (3) paying the extinction debt (mortality time lag) because of the deterioration of conditions (prevailing adverse effect of higher water input or more extreme variability of cold and warm conditions). A flawless approach to these hypotheses would be based on a sowing experiment^34^. However, severe conditions on the edge of vegetation does not allow every-year generative reproduction or seedling establishment. In fact, such propitious seasons are rather rare and the populations at the upper range margins are often demographic sinks^35^, i.e. established and maintained thanks to seeds dispersed from lower altitudes^6^,^36^. Therefore, if the experimental seeds were sown with no success, it could just mean that we failed to match the rarely occurring suitable year. Thus, we adopted an alternative approach, and conducted a transplant experiment to test our three hypotheses of species response. The survival/persistence of mature plants transplanted beyond their current range would suggest dispersal and/or recruitment limitation, and, accordingly, the hypothesis of habitat limitation would be rejected. If no plants survived, habitat limitation would be concluded to be the primary cause.

Growth in cold environments is primarily determined by temperature^6^, stressing the importance of sink-limitation; even if there are sufficient resources, the prevailing low temperatures slow their incorporation into new tissues as enzymatic systems run in suboptimal conditions. Consequently, alpine plants often have higher concentrations of nutrients in their tissues^37^. In contrast, there is some indication that at extreme elevations the pattern changes and low nutrient uptake can limit growth^38^; nutrient availability may play a greater role than previously thought. Fertilization was therefore included in the experiment to examine possible effects of nutrient limitation.

The main objectives of this study were to examine if (1) mature plants transplanted beyond their uppermost range margins are able to survive, (2) microclimate amelioration and nutrient addition support survival, and (3) particular functional traits are associated with better survival.

## Results

### Microclimate

**Figure 1:**
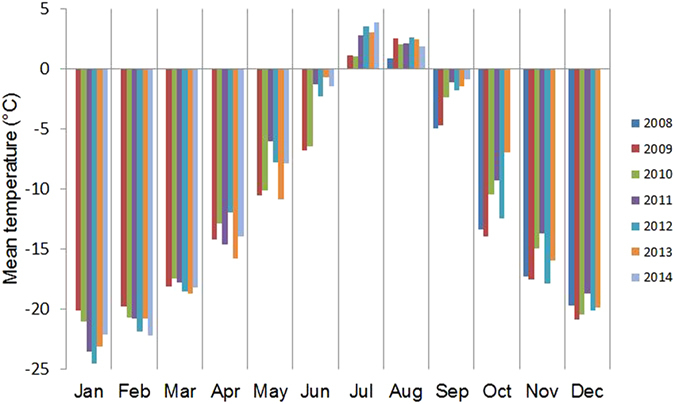
Mean monthly air temperature of Chamser Kangri Massif for period 2009–2014. Recorded 30 cm above ground ca. 1 km from experimental sites at 5900 m a.s.l. in hourly intervals.

Growing season length at the transplant sites decreased with elevation, and its year-to-year variation was considerable (Table 1). Temperature measurements conducted during August 2008–August 2014 at 5900 m showed significant increase in mean temperature of summer months, namely June (+1.23 °C per year), July (+0.59 °C per year), and September (+0.69 °C per year) (Fig. 1; Supplementary Table S1). The same trend was recorded for mean daily maximum temperature. No trends were recorded for mean temperature of winter months, except for a cooling in February (−0.38 °C per year).

### Survival of species

At the source site at 5750 m, all but four species (*Desideria pumila*, *Ladakiella klimesii*, *Stellaria decumbens*, *Waldheimia tridactylites*) survived across the treatments but with variable success (Table 2). At the edge site at 5900 m, with the exception of *Saussurea inversa* all species survived, but again the numbers of surviving individuals differed a lot. *Saxifraga cernua* showed the second highest survival rate here, with six flowering individuals. This is especially noteworthy, as the 5900 m site lay 60 metres above the species’ natural limit. At the beyond-range site at 6100 m, individuals of three species survived (*Saxifraga cernua*, *S. nanella* and *Poa attenuata*). Seven surviving individuals of these species were inside the enclosure, but there were also two individuals in unsheltered plots (both control and fertilized). These three species had the highest survival rates at all the sites. Since 2013, no individuals were surviving at the beyond-range site.

None of the surviving plants at 6100 m was bearing flowers in 2012. At 5900 m, 18 out of 51 surviving plants were flowering while only two individuals out of 43 (both *P. attenuata*) were flowering at 5750 m (Table 2). Growth analysis was mostly inconclusive due to low numbers of surviving individuals (Supplementary Table S2). However, plants at 6100 m generally decreased in size and height and had lower number of shoots in 2012 compared to 2010, while at 5900 m we recorded size gains and increasing numbers of shoots.

Species surviving at upper elevation had epigeogenous rhizome (p = 0.0002, p_corr_ = 0.005, R^2^adj = 0.77), lower δ13C (p = 0.008, p_corr_ = 0.18, R^2^adj = 0.51), higher C:N ratio (p = 0.02, p_corr_ = 0.446, R^2^adj = 0.41) and lower LPC (p = 0.0002, p_corr_ = 0.961, R^2^adj = 0.31).

### Effect of treatments

**Figure 2:**
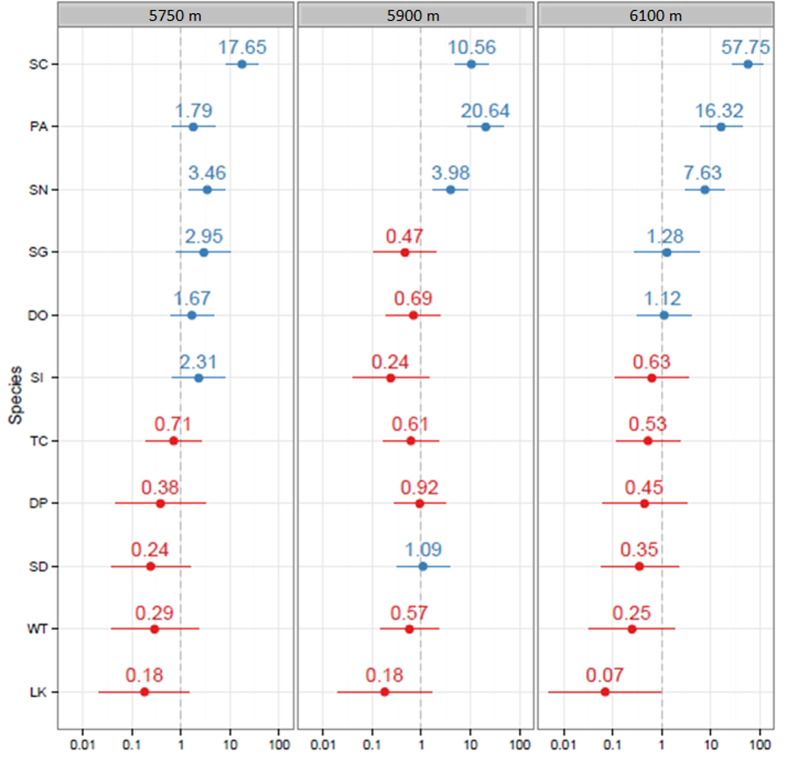
Survival of species-odds-ratios for random effect at species level for each elevation. Survival higher than expected at all elevations was consistently found for *Saxifraga cernua* (SC), *Poa attenuata* (PA) and *Saxifraga nanella* (SN). Low survival rate of *Ladakiella klimesii* (LK) was evident in all treatments. Other species showed an intermediate or inconsistent response to experimental transplantation along the elevation gradient (DO–*Draba oreades*, DP–*Desideria pumila*, SD–*Stellaria decumbens*, SG–*Saussurea glacialis*, SI–*Saussurea inversa*, TC–*Thylacospermum caespitosum*, WT–*Waldheimia tridactylites*).

**Figure 3:**
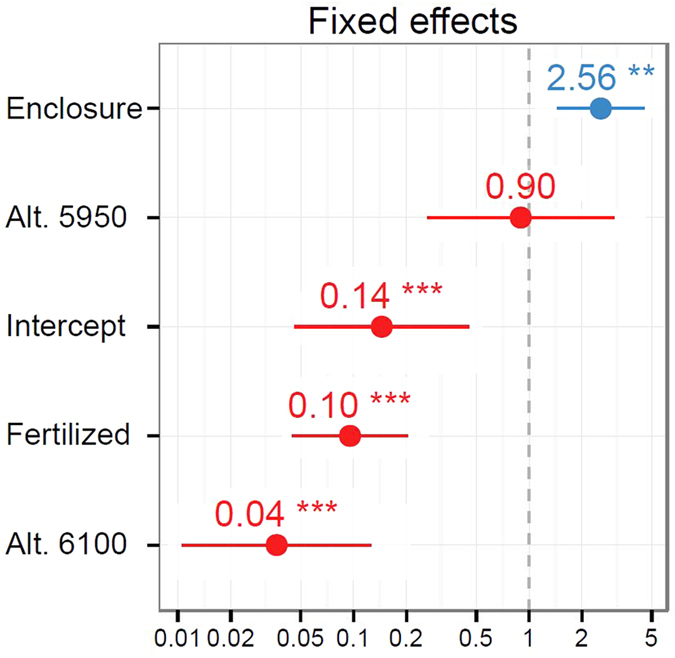
Treatment effects odd-ratios on plant survival. Stone enclosure (Enclosure) shows a significant positive effect, while fertilization had a strong negative effect. Survival at edge site (Alt. 5900) was indistinguishable from source site, effect of transplantation to beyond-range site was solely negative (Alt. 6100).

Site elevation affected survival (χ^2^ = 35.310 on 2 d.f., p < 0.001; Fig. 2). Survival at the edge site was not significantly different from that at the source site (OR = 0.9, z = −0.177, p = 0.859), while survival at the beyond-range site was considerably lower (OR = 0.04, z = −5.224, p < 0.001). Overall, stone enclosures had a positive effect on plant survival (OR = 2.56; χ^2^ = 10.527 on 1 d.f., p = 0.001; Fig. 3). The application of fertilizer affected plant survival negatively (OR = 0.10; χ^2^ = 37.093 on 1 d.f., p < 0.001; Fig. 3).

**Figure 4:**
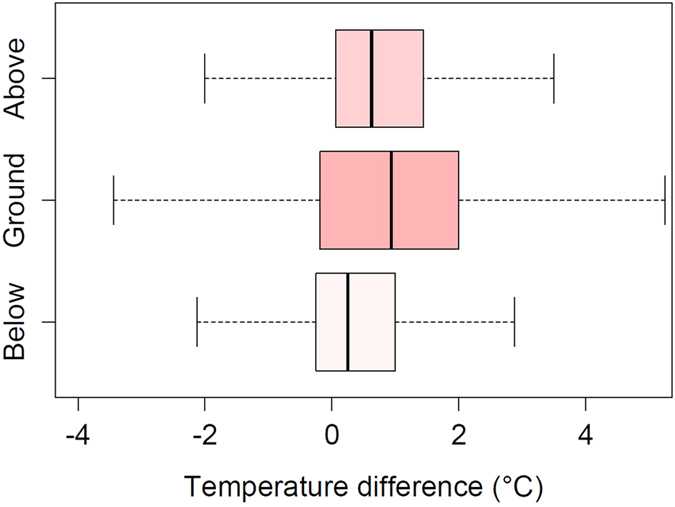
Effect of stone enclosure on microclimate. Differences between sheltered (stone enclosures) and unsheltered plots in below ground (−5 cm), ground, above ground (10 cm) temperatures on the edge-site at 5900 m in 2010. Note that stone enclosures increased the temperature in all three cases.

At 5900 m, the mean annual temperature of the ground inside the stone enclosure was 0.94 K higher compared to control plot (Fig. 4). The increase in soil and near-ground temperatures was less pronounced (0.25 and 0.63 K, respectively).

## Discussion

Nine mature individuals of three species survived three years above the regional elevation limit of continuous vegetation. Although far from thriving, those nine plants persisted for three years after their transplantation and went extinct only then. A three-year-long period of persistence is sufficient enough to show that the site was habitable and would imply that some of the studied species have a potential for upward migration. The upward migration, however, will depend on seed production in source populations^39^, species′ dispersal capacities^2^,^40^ and on the suitability of habitats for seedling establishment^41^,^42^. Therefore, the response of species to climate change at the upper limit will be species-specific^43^,^44^.

Our finding contrasts with the results of an earlier transplant experiment from the region^8^, performed in 2001, where plants were able to survive only at the elevation of their natural maximum occurrence. Klimeš and Doležal^8^ concluded that this elevation (~6000 m) was limiting for vegetation and the higher altitudes were uninhabitable for vascular plants due to ecophysiological constraints resulting from the non-existence of suitable habitats. It is likely that the three-year-long survival on our beyond-range site is connected to the warming of the region. Large-scale warming trends and predictions announcing further warming seem to be univocal for this region in a broad sense (NW Himalaya, W Tibet), and the *in-situ* temperature measurements taken were in accordance with these prognoses. The mean temperature of summer months keeps rising and this fact alone would support an upward shift of vegetation, similar to adjacent parts of the Himalayas^45^. The stone enclosures simulated the natural situation where plants in these extreme elevations occur in the shelter of larger stones which provide similar benefits. Nevertheless, the plants in our study were also able to survive in the unsheltered plots which shows their ability to survive on exposed ground without shelter. Interestingly, in 2010, we discovered an isolated outpost site at 6150 m colonized by five species of vascular plants (Table 2). This site was microclimatically exceptional-facing southwest and being sheltered by large stones accumulating heat, the site had microclimate similar to sites in substantially lower elevations, e.g. the edge transplant site at 5900 m (Table 1). This finding nicely illustrates the fact that the elevation per se is not the key driver behind the upper limit of vascular plants^6^.

The fertilizer amendment decreased the probability of survival, even if the nutrient release in this arid climate was exceptionally slow and the tablets were not yet fully dissolved after three years in 2012. This supports the idea of sink-limitation in high-altitude plants which are unable to utilize extra nutrients due to limiting effects of low temperatures on enzymatic systems^6^.

**Figure 5:**
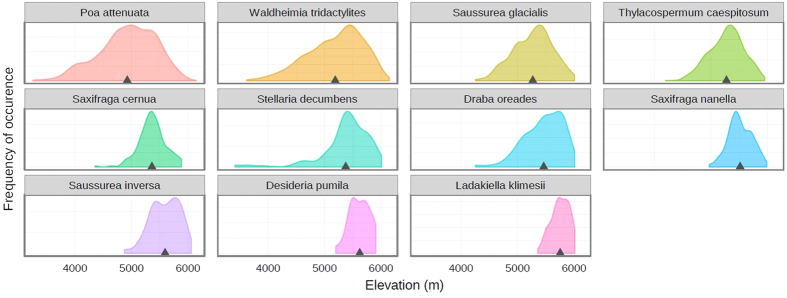
Elevational ranges of 11 species transplanted beyond their local elevational maximum. Species are arranged according to the increasing elevation optimum. Black triangles depict the mean elevation of localities where the species is present.

Regardless of the elevation, the best surviving species were *Poa attenuata*, *Saxifraga cernua* and *S. nanella*. These three species have in common their adventive root system and epigeogenous rhizomes^46^. Under the challenging condition of everyday freezing and thawing of the substrate, such a root system may prove advantageous in comparison to a tap root. *Saxifraga cernua* has the potential for fast, vegetative spreading due to its detachable bulbils located in leaf-axils. In one case, it established a new plantlet from the detached bulbil on the edge-site in 2010, moreover two detached bulbils were found on the beyond-range site in 2011. This fact is especially interesting given that *Saxifraga cernua* had the natural upper limit at the lowest elevation (5890 m; Fig. 5) of all of the transplanted species and thus seems more dispersal-limited at this elevation than others. The only species which bore flowers in 2012 at the edge-site were likewise *Poa attenuata*, *Saxifraga cernua* and *S. nanella*, a fact which illustrates their success. However, no plants flowered at the beyond-range site.

Our results bring new insight into the minimum heat requirements of vascular plants. So far, the lowest sum of heat recorded at a place with vascular plants was 121 degreedays above 0 °C in the root-zone (Himalaya, 6030 m)^8^, followed by 178 degreedays reported from 4545 m in the Alps (Körner^7^). Klimeš and Doležal^8^ found the upper elevational limit of vascular plants (*Poa attenuata* and *Waldheimia tridactylites*) at 6030 m on the western slope of Chalung Peak, situated 10 km north of our study site; their measurements in 2002–2003 showed the growing season to be about 50 days long with seasonal mean root-zone temperature as low as 1.7 °C. The presence of vascular plants at this locality, however, was not confirmed in 2014, and the site was completely covered by a thick layer of frozen snow during the whole vegetation season in 2015. The coldest sites in our experiment, where vascular plants naturally occurred, were those at 5900 m and 6150 m; both sites accumulated 163 degreedays above 0 °C. This suggest that the minimum growing season length for persistent vascular plant life is about 60 days, with seasonal mean root-zone temperature 2–3 °C^7^, which results in the sum of heat greater than ca. 160 degreedays above 0 °C. Interestingly, both uppermost plant localities with available microclimatic data (Chalung Peak 6030 m and Shukule Peak 6150 m) have in common a period of at least four weeks without frost in the root-zone, despite their difference in absolute elevations and seasonal mean temperature (1.7 °C vs 2.8 °C). This suggests that the root-zone temperature is more important determinant of upper elevational limit of vascular plants than other commonly used climatic measures, e.g. mean ambient temperature. The freezing of the soil is more critical for vascular plant survival than the above-ground frost because rhizodermis of roots represents a less effective barrier against ice-nucleation when in contact with external soil ice^47^.

The variation in the upper limit among species was found to be fairly low, not broader than ca. 100 m of elevation. A similar finding was reported by Dvorský *et al.*^10^ who focused on the upper elevation limits of subnival species in the region; 16 species out of the 24 reaching above 5800 m had their upper limit within 100 m of 6000 m, with a steep decline towards the higher elevations. In our experiment a relatively small increase in elevation from 5900 to 6100 m limited the survival of most transplanted plants. Such a sharp boundary reflects a strong limitation through temperature-related constraints at these range margins, resulting in asymmetric (skewed) distribution curves^48^. Additionally, the steep vegetation decline at ca. 6000 m is further reinforced by a physical constraint caused by terrain morphology. The elevation of 6000 m is right at the point where the gentle plateau slopes steepen; where the protruding windswept peaks start raising. This determines an abrupt change in microclimate. While on the gentle slopes the temperature is steadily decreasing, there is a sudden jump at 6000 m. Indeed, the adiabatic lapse rate of atmospheric temperature, which in Himalaya ranges 0.5–1 K per 100 m from humid to arid regions^6^, corresponds relatively well to the overall difference in mean temperature of the vegetation season of the transect. However, between 5750 and 5900 m sites the temperature gradient is rather moderate (0.6 K per 100 m in 2009 and only 0.33 K per 100 m in 2010), while the difference between 5900 m and 6100 m was greater than expected (1.0 and 1.5 K per 100 m in 2009 and 2010, respectively) and can be ascribed to the glacier proximity and increased exposure to strong winds at the upper site. Such strong microclimate deterioration was also noticed by Klimeš and Doležal^8^, who inferred from their measurements that the difference in the vegetation season length between their transplant sites at 6030 m and 6160 m was twofold, together with frequent freezing of the soil on the upper site. Another reason for the sharp upper limit is that the proportion of patches with suitable substrate compared to uninhabitable screes considerably drops, so that the distances for dispersal are larger and the overall probability of diaspores to reach a suitable microsite decreases, a factor which can itself result in range limits if microsite colonization rates are lower than extinction rates^49^.

## Conclusion

In the first three years of our experiment (2009–2012), there were plants surviving beyond the regional upper limit of vegetation. This supports the hypothesis of dispersal and/or recruitment limitation. Significant warming, recorded *in-situ* during this period, very likely played an important role in the survival. The survival, however, was ecologically non-random process and species better adapted to repeated freezing and thawing of the soil survived significantly better. No species have survived above the actual vegetation limit since 2013, which we ascribe to the extreme snowfall in 2013. Thus, our experiment sheds light on the complex dynamics of vegetation at its absolute elevational limit where only a small shift in microclimate, e.g. caused by an episodic climatic event, determines whether a population survives or dies off. Our results show that slope exposure and micro-topography are crucial for a successful colonisation and, on the local scale, may be more important than elevation as such.

We conclude that the current elevational limit of vascular plants in the region is maintained by the minimum heat requirements of species, accompanied by a combination of limitations in seed production, dispersal and recruitment. Episodic climatic events also play a great role because of their immediate impact. Considerable warming of the region as well as individualistic responses of the species support the idea that the composition of high-elevation plant communities might change in the future, though the change is not presumed to be fast, given the slow-growing nature of species, their ability to persist even under advert conditions, and the limitations in propagation.

## Methods

### Study region

The study was conducted in Changthang region in East Ladakh, Jammu & Kashmir state, NW India. It represents the southwesternmost extension of the Tibetan Plateau with relatively gentle slopes and several peaks exceeding 6500 m. Experiments were performed on the north slope of Chamser Kangri Peak (6622 m a.s.l.), located east of Tsomoriri Lake at the end of the Lupgo valley (N 32° 59.933′ E 78° 26.601′). The climate is arid (ca. 100 mm yr^−1^), most precipitation falls during summer, and above ca. 5000 m practically only snowfall occurs. Winter precipitation is erratic; snow pack is usually thin and discontinuous within the region^50^. The temperature regime is characterized by a large diurnal range^10^. Bedrock is composed of gneiss; soils are characterised by their relatively fine-grained structure, high pH (7.5–9), relatively high concentrations of macronutrients (Supplementary Table S3), and with low organic matter content^51^. High pH values of the substrate are caused by alkaline salts which are widespread in the region. Biological soil crusts are a typical feature on the surface of subnival soils^52^. Vegetation is continuous, although sparsely scattered, up to 5950–6000 m, but isolated micropopulations on exceptionally favourable microsites can be found even higher^10^.

### Experiment

In 2008–2009 we mapped the upper elevation range of subnival species between Chamser Kangri and Shukule Peaks. In addition, we compared our findings with the results of floristic mapping carried out in 2001–2003 within a wider region by our late colleague Leoš Klimeš.

On 12 August 2009, we transplanted 11 common subnival species (Table 2) from the core of the subnival zone (5750 m) to a nearby site at the same elevation (source site, control), to the edge of the vegetation range (edge site, 5900 m) and beyond it (beyond-range site, 6100 m; Fig. 1). The target species represented different growth and functional types of plants^46^ (loose to compact cushions, rhizomatous, rosette or turf plants, clonal and non-clonal). They were randomly selected from the core of the subnival zone, removed as carefully as possible with the soil attached to the roots and transplanted on the same day.

**Figure 6:**
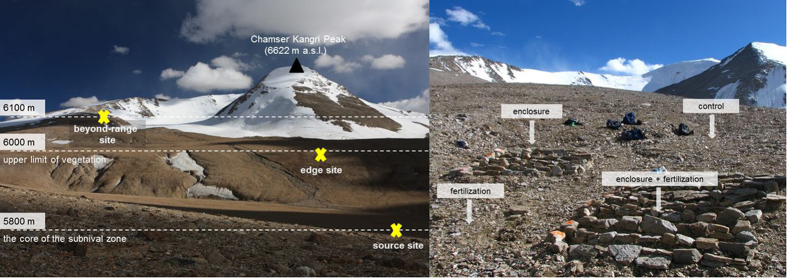
Slope with experimental sites (left), treatment plots within the edge transplant site at 5900 m (right).

We applied two treatments in full factorial design, resulting in four plots at each of the three transplants sites: 1) control plot, 2) stone enclosure, 3) fertilizer addition and 4) stone enclosure and fertilizer addition (Supplementary Fig. S4). The two plots with fertilizers were never located upslope from the non-fertilized plots to avoid contamination. There were 8 rows within each plot, each row contained 11 species randomly mixed, with individuals planted ca. 15 cm apart. In total, we transplanted 1056 individuals (3 altitudes × 4 plots × 8 rows × 11 species). We built enclosures made of stones ca. 40 cm in size (Fig. 6) in an attempt to ameliorate the conditions, specifically to increase the temperature and protect against wind, and buffer temperature oscillations. Silvamix® (Ecolab Znojmo, Czech Republic) fertiliser tablets with slow nutrient release (NPK with trace elements) were used. One tablet was placed ca. 3 cm from each plant, resulting in a total amount of 80–100 kg N ha^−1^ yr^−1^.

Microclimatic loggers were placed at each site. Air humidity and soil surface temperature were recorded by R3120 loggers (Comet System, Brno, Czech Republic). Two iButton® loggers DS1921G (Maxim Integrated, San Jose, USA) recorded soil temperature 5 cm below the surface; one logger was inside the stone enclosure, the other one in the control plot. The air temperature at 10 cm above soil surface was recorded by TMS loggers (TOMST®, Prague, Czech Republic). In addition, HOBO® logger (Onset, Bourne, USA) was recording the air temperature and relative air humidity for the duration of the experiment (2009–2015); it was placed at 5900 m, ca. 1 km off the experimental sites 30 cm above ground, shaded. Due to an unfortunate series of troubles with microclimatic loggers (discontinuous records) which made basic comparisons among sites and treatments impossible, we had to introduce other TMS loggers in 2013 (source site at 5750 m, edge site enclosure at 5900 m, beyond-range site enclosure at 6100 m). Growing season length was calculated as number of days with mean soil temperature above zero^7^,^8^.

At each transplanted plant, we were measuring annual growth (size of leaf-rosette, height, number of shoots, number of flowers) for three years at the end of vegetation season until the final assessment in September 2012. From 2013 to 2015, we made only cursory examinations of the transplant sites, particularly to check the beyond-range site for surviving individuals.

### Functional differences between species

We used a database of functional traits of high-altitude plants in Eastern Ladakh by Chlumská^53^ (with the methods of data collection therein; see also Cornelissen *et al.*^54^) in order to reveal if any of the traits can explain differenes in survival of the transplanted species. We employed plant height, growth form^46^, leaf and stem dry matter content (LDMC and StDMC), C:N ratio, seed mass, leaf nitrogen and phosphorus concentrations (LNC, LPC), leaf δ^13^C, root nitrogen and phosphorus concentrations (RNC, RPC), non-structural soluble carbohydrates (NSC; starch, fructans, raffinose family oligosaccharides, simple sugars and sugar alcohols). The traits were measured in a minimum of 10 individuals (up to 50 for some species) from various elevations and the average values were calculated per species.

### Data analysis

To explore the general effects of experimental treatments, we analysed the data on transplanted plants by generalized mixed effect models. Mortality in the first year of experiment could be induced by direct plant damage due to transplantation, therefore we ran analyses only on second-year survival. We used the *glmer* function with logit link function from R package *lme4*^55^. We tested the significance of model terms by type II Wald χ^2^ test. Wald z statistics were used to test the significance of treatment levels. We obtained effect odds ratios (OR) and visualized models by *sjp.glmer* function from R package *sjPlot* (Lüdecke 2015). We tested the effects of experimental treatments (site elevation, enclosure, fertilization) on survival as fixed effects, while species identity was considered as a random effect. We expected a species-specific response to experimental treatments beyond their elevational range, therefore we used the Akaike information criterion (AIC) to compare the performance of the model with only random intercepts for each species and the model with random effects on each elevation level for each species. The model with lower AIC was selected. To interpret these random effects, we searched for correlation between species functional traits and corresponding random effects. We applied Bonferroni correction to model ANOVA p-values to correct for multiple testing (p_corr_). The analyses were performed in R (R Development Core Team 2015).

## Additional Information

**How to cite this article**: Dvorský, M. *et al.* Gardening in the zone of death: an experimental assessment of the absolute elevation limit of vascular plants. *Sci. Rep.* **6**, 24440; doi: 10.1038/srep24440 (2016).

## Supplementary Material

Supplementary Information

## Figures and Tables

**Table 1:** Microclimatic characteristics of sites at contrasting elevations in Changthang, eastern Ladakh.

Site		Source			Edge			Beyond-range			Outpost			Klimeš & Doležal^8^			
Treatment		Control			Enclosure			Enclosure			–			–		–	
Elevation [m a.s.l.]		5750*			5900*			6100			6150*			6030*		6160	
Exposure		West			Northwest			Northwest			Southwest			West		West	
Temperature [°C]**		Soil	Surface	Air	Soil	Surface	Air	Soil	Surface	Air	Soil	Surface	Air	Soil	Air	Soil	Air
Annual mean		−6.9	−8.2	−9.9	−8.7	−9.6	−11.1	−10.3	−10.8	−12.2	−8.1	−9.9	−11.6	−8.2	−8.1	−11.6	−12.2
Absolute maximum		16.1	23.3	21.9	12.9	22.8	22.5	9.8	15.4	20.3	12.0	16.5	23.8	11.0	17.5	7.0	14.9
Absolute minimum		−22.3	−29.9	−34.4	−22.5	−28.4	−38.9	−26.6	−29.1	−40.6	−24.3	−31.0	−38.2	−18.1	−18.1	−26.1	−31.3
Maximum daily range	Year	13.8	27.0	26.9	12.8	26.1	29.7	11.1	18.3	26.9	13.6	26.0	30.1	11.1	21.4	11.4	20.9
Growing season length [days] /Degree days >0 °C	2003									47/121		19/19					
	2010	92/251			70/268			55/95									
	2014	119/446			89/235			65/135			111/241						
	2015	88/270			71/163			45/48			89/163						

Temperature was recorded by TMS loggers 10 cm deep in the soil, on the surface, and 10 cm above the surface during 2009–2015 at hourly intervals. Klimeš and Doležal^8^ recorded temperature 5 cm deep in the soil and 10 cm above the surface during 2002–2003 at hourly intervals. Growing season is taken as a period with daily mean temperature of soil above 0 °C. Growing degree-days (above 0 °C) were calculated from hourly temperature recordings. *indicate sites where vascular plants naturally occurred, **values for the year 2014 (Source, Edge, Beyond-range, Outpost), and for the year 2003 (Klimeš & Doležal^8^).

**Table 2:** Transplanted species.

Species	Fam	Min alt	Max alt	Source site (5800 m)					Edge-site (5950 m)					Beyond-range site (6100 m)				
				C	F	E	EF	∑	C	F	E	EF	∑	C	F	E	EF	∑
*Desideria pumila* (Kurz) Al-Shehbaz	B	5380	5990	–	–	–	–	*0*	–	–	3	–	*3*	–	–	–	–	*0*
*Draba oreades* Schrenk	B	4800	6010	–	–	4	1	*5*	2	–	–	–	*2*	–	–	–	–	*0*
*Ladakiella klimesii* (Al–Shehbaz) D.A. German & Al–Shehbaz	B	5350	6150*	–	–	–	–	*0*	–	–	1	–	*1*	–	–	–	–	*0*
*Poa attenuata* Trin.	P	4540	6000	–	1	3(1)	1(1)	*5*	5(1)	1(1)	8(5)	3(3)	*17*	–	–	1	–	*1*
*Saussurea glacialis* Herder	A	4595	6150*	2	1	1	–	*4*	–	–	1	–	*1*	–	–	–	–	*0*
*Saussurea inversa* Raab–Straube	A	5250	6150*	3	–	1	–	*4*	–	–	–	–	*0*	–	–	–	–	*0*
*Saxifraga cernua* L.	S	4650	5890	7	1	8		*16*	2	–	7(3)	3(3)	*12*	1	1	4	–	*6*
*Saxifraga nanella* Engl. & Irmsch.	S	5150	5995	2	–	5	–	*7*	2	–	6(2)	–	*8*	–	–	2	–	*2*
*Stellaria decumbens* Edgew.	C	4560	6060	–	–	–	–	*0*	1	–	2	–	*3*	–	–	–	–	*0*
*Thylacospermum caespitosum* (Cambess.) Schischk.	C	4550	5960	1	–	1	–	*2*	2	–	–	–	*2*	–	–	–	–	*0*
*Waldheimia tridactylites* Kar. & Kir.	A	4820	6150*	–	–	–	–	*0*	2	–	–	–	*2*	–	–	–	–	*0*
			total	15	3	20	1	***43***	11	0	7	0	***51***	1	1	7	0	***9***

Fam-family (A-Asteraceae, B-Brassicaceae, C-Caryophyllaceae, P-Poaceae, S-Saxifragaceae), distribution in East Ladakh (Min alt-low elevation limit, Max alt-upper elevation limit; *indicate species found at a remote outpost beyond the range of continuous vegetation), number of surviving individuals in 2012 in respective treatment plots (maximum number is 8; number in brackets indicate number of individuals flowering in 2012, otherwise there were no flowering individuals; C-control plot, F-fertilised plot, E-enclosure, EF-enclosure with fertilisation, ∑-sum of surviving species in all plots).
