# Supplementary material for: Gardening in the zone of death: an experimental assessment of the absolute elevation limit of vascular plants
Source: Sci Rep. 2016 Apr 13;6:24440. doi: 10.1038/srep24440 (PMC4829891; doi:10.1038/srep24440)
Supplement: Supplementary Information [file srep24440-s1.pdf]

Supplementary information

Gardening in the zone of death: an experimental assessment of the absolute elevation limit of vascular plants.

Miroslav Dvorský[1,2*], Zuzana Chlumská[1,2], Jan Altman[1,2], Kateřina Čapková[3], Klára Řeháková[1], Martin Macek[4,5], Martin Kopecký[6], Pierre Liancourt[1], Jiří Doležal[1,2]

Supplementary Table S1. Trends in mean daily temperature of respective months. Linear regression equation, regression coefficient (R^2) and level of significance (P; *** P< 0.001; ** P< 0.01; * P< 0.05) are shown.

Month	Linear Regression	R^2	P
January	y = -0.4803x + 943.73	0.307	0.254
February	y = -0.3852x + 753.92	0.67	0.046*
March	y = -0.1389x + 261.3	0.308	0.253
April	y = -0.1452x + 278.15	0.041	0.699
May	y = 0.2697x - 551.29	0.068	0.618
June	y = 1.2272x - 2471.8	0.709	0.035*
July	y = 0.5909x - 1186	0.821	0.013*
August	y = 0.1172x - 233.65	0.184	0.337
September	y = 0.69x - 1390	0.795	0.007**
October	y = 1.0713x - 2165	0.562	0.085
November	y = 0.2028x - 423.9	0.052	0.665
December	y = 0.0861x - 192.97	0.047	0.68

Supplementary Table S2. Growth and flowering of plants on the transplant sites at 5950 and 6100 m a. s. l. Values represent per cent change in size, number of shoots, height, and change in the number of flowering shoots from 2010 to 2012. Size (%) – per cent change in the size (length*width of the basal part of an individual) of plants; Shoot (%) – per cent change in the total number of shoots; Height (%) – per cent change in the height of the highest sterile shoot; Flowering shoots – change in the number of flowering shoots (e.g. 0 = the plant never flowered or the number of flowers remained the same; 1 = the plant had one more flowering shoot in 2012 compared to 2010; -2 = the plant had two flowering shoots fewer in 2012 compared to 2010). p – significance from one sample t-test (H_0 = 100 %, i.e. plant didn´t grow), * $p< 0.05$, ** $p<0.01$, *** $p<0.001$, ns – non-significant. No – number of individuals.

	Change from 2010 to 2012											
**treatment**	**Size (%)**	**p**	**No**	**Shoots (%)**	**p**	**No**	**Height (%)**	**p**	**No**	**Flowering shoots**	**p**	**No**
**5900 m a. s. l.**	**132.6**	**ns**	**37**	**74.2**	**.**	**32**	**122.6**	**ns**	**48**	**1.3**	**.**	**59**
**fertilized**	**148.1**		**1**	**40.0**		**1**	**285.7**		**1**	**6.0**		**1**
Poa attenuata	148.1		1	40.0		1	285.7		1	6.0		1
**control**	**51.7**	**.**	**12**	**39.1**	**	**10**	**106.3**	**ns**	**15**	**0.1**	**ns**	**24**
Poa attenuata	33.8	.	4	44.0	ns	2	75.0	ns	5	0.5	ns	8
Draba oreades	11.9		1	16.0	.	2	125.0	ns	2	0.0		2
Saxifraga cernua	130.3	ns	2	95.8	ns	2	145.0	ns	2	-0.7	ns	3
Stellaria decumbens	55.7		1				250.0		1	0.0		2
Saxifraga nanella	42.5	ns	2	16.6	ns	2	100.0		2	0.0		4
Thylacospermum caespitosum	35.7	ns	2				50.0		1	0.0		2
Waldheimia tridactylites				23.2	ns	2	90.0	ns	2	0.0		2
**enclosure**	**99.9**	**ns**	**18**	**92.6**	**ns**	**18**	**123.7**	**ns**	**26**	**0.6**	**ns**	**28**
Poa attenuata	108.4	ns	8	300.0		1	116.4	ns	8	3.0	.	8

Ladakiella klimesii										0.0		1
Desideria pumila				33.0	ns	3	150.0	ns	3	-2.0	ns	3
Saxifraga cernua	174.9	ns	4	135.7	ns	7	152.8	ns	7	-0.1	ns	7
Stellaria decumbens	30.8	ns	2				150.0		1	0.0		2
Saussurea glacialis	48.0		1	100.0		1	66.7		1	0.0		1
Saxifraga nanella	40.9	ns	3	36.4	*	6	91.7	ns	6	0.2	ns	6
**fertilized and enclosure**	**389.6**	**ns**	**6**	**91.4**	**ns**	**3**	**131.2**	**ns**	**6**	**8.5**	**ns**	**6**
Poa attenuata	171.5	ns	3				69.7	ns	3	13.3	ns	3
Saxifraga cernua	607.7	ns	3	91.4	ns	3	192.7	ns	3	3.7	*	3
**6100 m a. s. l.**	**16.0**	*****	**4**	**48.1**	*****	**9**	**34.5**	******	**6**	**-0.1**	**ns**	**9**
**fertilized**	**6.7**		**1**	**28.6**		**1**	**20.0**		**1**	**-1.0**		**1**
Saxifraga cernua	6.7		1	28.6		1	20.0		1	-1.0		1
**control**				**50.0**		**1**				**0.0**		**1**
Saxifraga cernua				50.0		1				0.0		1
**enclosure**	**19.2**	**.**	**3**	**50.6**	**.**	**7**	**37.4**	*****	**5**	**0.0**		**7**
Poa attenuata	0.7		1	15.0		1	46.7		1	0.0		1
Saxifraga cernua	53.3		1	75.6	ns	4	24.5	*	3	0.0		4
Saxifraga nanella	3.5		1	18.3	.	2	66.7		1	0.0		2

Supplementary Table S3. Soil qualities at transplant sites. Numbers represent averages from 12 soil samples per site. Soil texture means the proportion (%) of soil particles bigger than 0.5 mm.

	5800 m	5950 m	6100 m
Soil texture	2.7	6.6	4.4
pH	7.8	8.7	9.1
TN [mg/kg]	1906	1072	941
P-PO_4^{3-} [mg/kg]	44	227	172

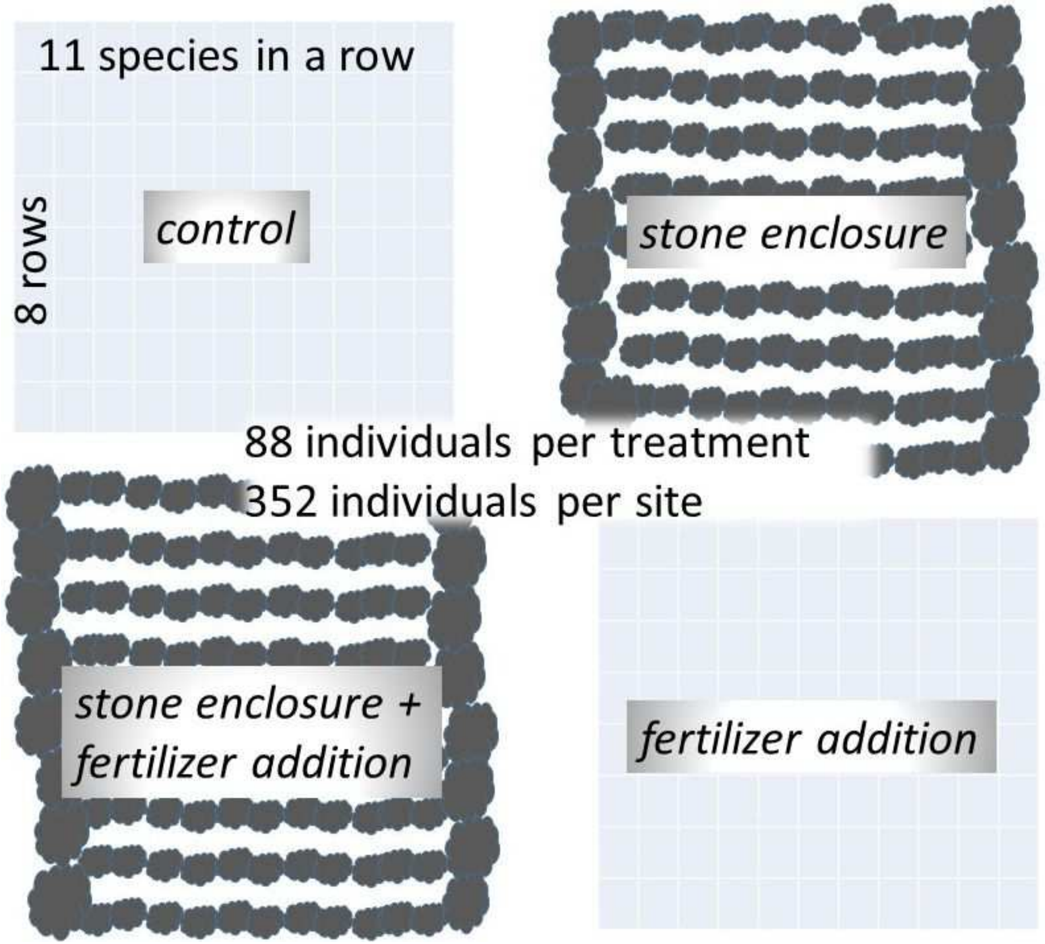

Supplementary Figure S4. A top view sketch of the experimental arrangement.
